# The Impact of Green Credit on the Green Innovation Level of Heavy-Polluting Enterprises—Evidence from China

**DOI:** 10.3390/ijerph19020650

**Published:** 2022-01-06

**Authors:** Zhifeng Zhang, Hongyan Duan, Shuangshuang Shan, Qingzhi Liu, Wenhui Geng

**Affiliations:** 1 School of Economics, Qingdao University, Qingdao 266071, China; sasha_china@hotmail.com (Z.Z.); wh.geng@outlook.com (W.G.); 2 Department of Economics, The University of Sheffield, Sheffield S10 2TN, UK; hduan4@sheffield.ac.uk; 3 School of Foreign Language Education, Qingdao University, Qingdao 266071, China; 4 Department of Economics, Shandong University of Science and Technology, Taian 271019, China

**Keywords:** green credit, heavy pollution enterprise, green innovation, investment efficiency, financing constraint, difference-in-difference model

## Abstract

This article uses the “Green Credit Guidelines” promulgated in 2012 as an example to construct a quasi-natural experiment and uses the double difference method to test the impact of the implementation of the “Green Credit Guidelines” on the green innovation activities of heavy-polluting enterprises. The study found that, in comparison to non-heavy polluting enterprises, the implementation of green credit policies inhibited the green innovation of all heavy-polluting enterprises. In the analysis of heterogeneity, this restraint effect did not differ significantly due to the nature of property rights and the company’s size. The mechanism test showed that green credit policy limits the efficiency of business investment and increases the cost of financing business debt. Eliminating corporate credit financing, particularly long-term borrowing, negatively impacts the green innovation behavior of listed companies.

## 1. Introduction

Over the last few years, due to the impact of greenhouse gas, particularly carbon dioxide emissions, global warming and climate change issues have become increasingly severe, attracting the attention of many countries. Green technological innovation is considered to be an important means to achieve sustainable development. Technological change, financial liberalization and globalization have reinforced the boundaries among countries on technological capabilities and competitiveness [1]. Within the development of green management around the world, industry competition has become more complex and uncertain. The development of the majority of products and technologies is evolving towards a green structure. When planning commercial strategies, taking environmental impact into consideration has led to major changes in the social system and competition area [2,3]. As the main consumer of resources and energy, heavy-polluting enterprises are the main producers of environmental pollution [4]. Heavy-polluting industries represented by electricity and steel are pillar industries that drive economic development. However, they are also the main culprits of environmental problems [5]. Heavy-polluting companies should take active social responsibility and implement the concept of developing environmental protection. In the process of green transformation, green innovation should serve as a new perspective, product and management system for addressing environmental problems. The authors of [6,7] pointed out that research into environmental innovation is still in its infancy. Therefore, focusing on enterprises in heavy-polluting industries, the relationship between green credit policies and green technological innovation remains to be further investigated.

Through the guidance of green technology innovation policies, companies could improve resource utilization efficiency and achieve a win–win development of social energy conservation and emission reduction [8,9]. The government typically implements green technology innovation policies based on the following two elements: environmental monitoring and funding restrictions. Ref. [10] pioneered empirical research in this area and used manufacturing data in the United States to confirm that environmental regulation could, to some extent, promote companies’ innovation. By limiting credit allocation and support to highly polluting and energy-consuming enterprises, more loan funds could be directed to environmentally friendly projects [11]. Theoretically, the Porter hypothesis suggests that a reasonable and strict environmental policy has a compensatory effect, prompting firms to internalize the pollution cost and improve innovation ability. However, green innovation is characterized by high inputs, high risks, long lead times and strong environmental externalities [12]. There is undoubtedly a need for reasonable and effective market intervention by the government. The government should take relevant policy measures to promote the green innovation. It is evident that the question of how to promote green transformation of enterprises through the promotion of technological innovation is an issue to be urgently explored.

Before the implementation of green credit in China, the International Finance Corporation (IFC) first proposed the concept of green credit in 2002, the “Equator Principles”. The “Equator Principles” aims to evaluate the environmental and social risks in project financing. To reduce pollution emissions and support high-quality economic development, the Chinese government announced a series of green finance policies aimed at reducing environmental degradation. Green credit is one of the most important green financial instruments implemented by the government. Financial institutions are encouraged to direct capital to cleaner production [13]. The “Opinions on Implementing Environmental Protection Policies and Regulations to Prevent Credit Risks”, issued in 2007, combined environmental regulation with credit regulation. This document made corporate environmental compliance one of the prerequisites for loan approval. However, due to the lack of clear implementation rules, the green credit policy is still in the theoretical stage. In 2012, the “Green Credit Guidelines” (GCP) were introduced, the first specific guidelines for financial institutions to carry out green credit. At present, some traditional heavy industries are still an important part of the national economy. Simply shutting down a large number of heavily polluting enterprises may lead to a shock in industrial development, unemployment and economic growth. Consequently, it is very important to find a way that would protect the environment and maintain stable economic development. In view of this, this article studies the impact of green credit on the level of green innovation of heavy-polluting enterprises.

The objective of this paper is to analyze the effectiveness of green credit policies. This article therefore draws up an econometric model. Variables include the level of green innovation, the effectiveness of investments, the level of debt financing and other factors. This study examines the impact of green credit policies on the level of green innovation of high-polluting companies. The marginal contributions of this article are as follows: First, concerning the effects of green credit policies, this article focuses on samples of highly polluting firms. This study will provide a practical basis for further investigation. Second, the green credit policy plays an important role in environmental oversight. Previous research on green credit policies has been subject to numerous omissions and shortcomings. However, this paper should not only look at the effects of green credit policies, but also discuss the mechanism of other possible factors which may work. Third, this paper enriches the research topics related to environmental regulation and pollution reduction. On the one hand, there is a lack of research on the level of green innovation in heavy polluters. On the other hand, this paper fully considers the problem of policy failure under the conditions of “strong government and weak society”, and provides a research basis for the full implementation of pollution reduction policies.

## 2. Literature Review and Theoretical Hypothesis

### 2.1. Literature Review

In recent years, in the face of an increasingly severe ecological environment, the Chinese government has paid more attention to the use of financial products in environmental governance and introduced a series of policies in the field of green credit. The subject of this paper is the impact of the implementation of green credit policies on the green innovation capacity of heavily polluting enterprises. What the green credit policy is actually trying to achieve is an improvement in society’s pollution reduction results. The improvement of the green innovation capacity of heavy polluters is only one path to achieving improved environmental performance of enterprises. As the indicators of the results of pollution reduction by enterprises have only been emphasized in recent years, the indicators are cumbersome. And the indicators are not of a uniform form. This has caused difficulties in the collation of data. This paper therefore focuses its attention on the impact of the implementation of green credit policies on the green innovation capacity of heavily polluting enterprises.

Current research on green credit policy is mainly divided into two main topics: theoretical analysis and effect assessment. More specifically, early in the study research, researchers were most interested in the need to implement green credit policies and the barriers to implementation. Ref. [14] elaborated systematically on green credit’s connotation and strategic value. Guo [15] thoroughly analyzed the challenges of implementing green credit guidelines and proposed a series of practical suggestions. Furthermore, some studies have assessed the performance of commercial banks and financial institutions in the implementation of green credit principles through model building and empirical analysis [16].

With the deepening of the operation of the policy, researchers are gradually focusing on the quantitative evaluation of the application effect of the green credit policy. Furthermore, research on this subject can be discussed from both a macro and a micro perspective.

At the macroscopic impact level, scholars have conducted in-depth discussions on the impact of green credit on the overall environmental quality. The authors of [17] found that green credit can reduce environmental pollution through three intermediary channels: improving business performance, fostering business innovation, and promoting the modernization of industrial structures. Ref. [18] used the spatial Dubin model to analyze the impact of green credit on China’s green economy and its transmission mechanism. They found that this policy can not only improve the local green economy, but also present Spatial spillover effects, which can promote the development of the green economy in surrounding areas to a certain extent. Based on China’s provincial panel data, Zhang et al. [19] concluded that the green credit can reduce China’s carbon emissions by encouraging modernization of industrial structures and technological innovation. Ref. [20] also confirmed that green credit has a major impact on promoting green and sustainable development in China. At the same time, environmental pollution is closely related to the industrial structure [21]. Some researchers have investigated the relationship between green credit and the industrial system of China. Ref. [22] demonstrated that green credit has a significant positive impact on the modernization of industrial structures as a whole, and Shao et al. [23] examined the dynamic development relationship between green credit and the rationalization of industrial structures in an innovative way. It turns out that there are three stages of degree of coupling from the bottom up. Implementing the green credit policy will help guide the rational allocation of resources and promote the development of the industry.

At the micro-impact level, most existing research is conducted around the two main bodies of companies and banks. For commercial banks, academics focus primarily on the impact of green credit implementation on bank performance. Ref. [24] used the systematic GMM regression method to test the relationship between green credit and the sustainable competitiveness of commercial banks. They noted that green credit policies could improve the performance of the bank’s total assets. King and Levine [25] also indicated that green credit could indeed improve the bank’s operational performance by encouraging the upgrading of the credit structure of commercial banks and reducing credit risk. However, some scholars’ studies have reached different conclusions. Luo et al. [26] developed a comprehensive rating index for commercial bank capabilities using factor analysis. The study found that green credit has a significant impact on improving the overall competitive position of banks, but with the progressive implementation of policies, this promotional effect will tend to become weaker. Yin [27] estimated that green credit would weaken commercial banks’ short-term financial performance, particularly for small and medium-sized commercial banks.

At the enterprise level, most scholars evaluate the policy effect of green credit from enterprises’ investment and financing status. Liu et al. [28] concluded that green credit policy harms investment and the financial situation of high-pollution and energy-intensive companies. Lemmon and Roberts [29] also confirmed that the green credit policy could limit the bank’s credit support to heavily polluting enterprises, which affects the financing activities of the enterprises to a certain extent. Li et al. [30] found that green credit will reduce the scale of debt financing of heavily polluting companies and increase costs by constructing a double differential model. Similarly, Peng et al. [31] have also found that green credit policies impact the extent to which high-polluting companies finance their debt. Zhang et al. [32] believed that green credit policies could promote short-term financing of “two highs” enterprises but negatively affect long-term funding and investment behavior. In addition, some scholars choose to focus on the impact of the implementation of green credit on the green behavior of enterprises. Research by [33] proves that green credit policies can help to increase investment in environmental protection by polluting companies. Furthermore, this promotion will vary depending on the nature and scale of the enterprise and the level of commercialization in the region. Zhang et al. [34] found that implementing Green Credit Guidelines helps businesses invest in renewable energy, and this promotion is heterogeneous. In addition, there is considerable academic interest in whether green credit influences business development performance. They found that green credit can increase the value of new energy businesses in a sustainable and long-term way [35]. Also, it will reduce the corporate performance of heavily polluting companies [36].

An analysis of many pieces of literature shows that research on the political impact of green credit is abundant at the macro-economic level. Furthermore, from a microeconomic perspective, the discussion on the effect of this policy on the performance of banks and investment and corporate finance is also very comprehensive. The fundamental objective of implementing the green credit policy is to encourage enterprises to proactively develop production and operation while respecting the environment. Green innovation is clearly an essential element of green transformation and company modernization. Unfortunately, at this point, little literature focuses on the specific effects of green credit policy on green innovation and its impact mechanism, and there are some differences in the conclusions drawn by existing studies. Ling et al. [37] used a difference-in-difference model to empirically analyze that green credit could negatively affect R&D investment and innovation output of enterprises when taking long-term debt as an intermediary, and this constraint has nothing to do with ownership rights and corporate scale. Caragnano [11] came to the same conclusion in their research. They believe that this policy reduces the scale of credit and increases the cost of credit for heavily polluting enterprises, leading to the intensification of credit constraints. However, the study of [38] noted that the green credit policy positively affects the technological innovation of high-polluting companies, and with increased commercialization, this promotional effect will be more meaningful. Hong et al. [39] are also of the opinion that Green Credit can promote innovation in green technologies across businesses. Furthermore, this influence will be heterogeneous depending on the type of ownership of the enterprise and the size. Liu et al. [40] proved that the implementation of green credit existed in the Porter effect by adopting PSM-DID. Hu et al. [41] also reached a similar conclusion. Guo et al. [42] found that green credit policies can effectively promote green technology innovation in one area, but there is no space spillover effect.

To sum up, the analysis of the micro effect of green credit in the existing literature is relatively limited, and there is a lack of relevant discussion on whether green credit can induce enterprise innovation transformation. At the same time, whether the implementation of this policy can promote enterprise green innovation has yet to be unified. In terms of research content, the above papers pay more attention to the direct impact of green credit policies on enterprises and banks, and neglect to fully sort out the policy mechanism. This article attempts to make up for these shortcomings. Concerning research samples, the scope of research in the existing literature is broader, and there is a lack of research on specific industries. This article uses highly polluting Chinese companies as a research sample to analyze the effects of policies at the industry level. In terms of empirical research, this article adopts the difference-in-difference method, which is appropriate for analyzing policies implemented in different regions and at different times.

### 2.2. Theoretical Hypothess

#### 2.2.1. Green Credit Policy and Green Innovation Level

At present, there is no unified opinion in the academic circles on the impact of environmental supervision policies represented by the “Green Credit Guidelines” on the innovation capabilities of enterprises. Pigou proposed the “Pigou Tax” in 1932, establishing a precedent for environmental control. Subsequently, Coase criticized Pigou’s method of correcting externalities. This is due to the fact that the “Pigou tax” restricts economic choices and stresses the important role of property rights and property rights transactions in environmental monitoring. According to using data on firms in major European countries, Rubashkina et al. [43] found that environmental regulation had an important role in facilitating their innovation. Hong et al. [39] states that there is an upward trend in the level of green innovation among firms, and an upward trend after the enactment of green credit. But what role does green credit policy play in this? Does it actively contribute to the promotion of green innovation capacity? As an environmental economic policy, the effect of green credit on enterprise technological innovation is mostly centered on the theoretical mechanisms of “Following cost “and “Porter hypothesis “. Following cost effect is reflected in the fact that environmental regulations will increase the production cost and pollution control cost of the enterprise. This produces a crowding-out effect on R&D investment activities and reduces the productivity of enterprises. The Porter hypothesis focuses on the innovation compensation effect. The Porter hypothesis as a reasonable environmental regulation has a positive effect on encouraging companies’ innovation.

Whaller and Whitehead [44] argues that environmental regulatory policies limit the ability of firms to innovate technologically by increasing the cost of production and operation and limiting the flow of finance to less polluting innovation projects. Lenoard [45] argues that companies subject to stringent environmental regulations can lose domestic and international market share and face increased investment costs due to environmental regulations. Consequently, some companies will reorganize production and investment in areas where monitoring is weak, which does not have a good impact on the environment. Yuan and Xiang [46] used data from Chinese manufacturers to conclude that rising pollution control costs inhibited companies’ innovation production. Kneller and Manderson [47] used environmental protection expenses to assess environmental regulations. They concluded that environmental law had not increased R&D investment in the UK manufacturing sector. The reason is that even though environmental regulation has increased investment in environmental R&D, it has been predatory. Shi et al. [48] estimated the impact of China’s pilot carbon emissions trading policy on business innovation outcomes and concluded that this policy significantly undermined the innovation of regulated and non-regulated firms. Based on the above analysis, the following assumption is proposed:

**Hypothesis** **1** **(H1).** *Following the promulgation of the “Green Credit Guidelines” in relation to non-polluting companies, highly polluting companies’ level of green innovation decreased considerably.*

#### 2.2.2. The Moderating Effect of Enterprise Investment Efficiency

**Figure 1:**
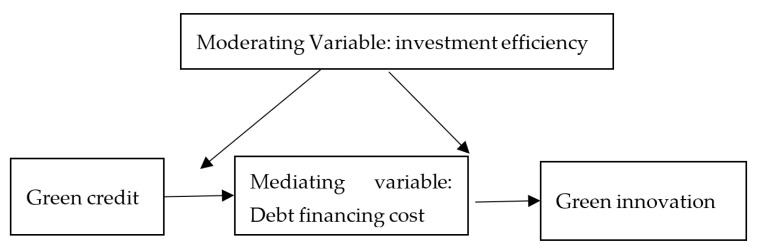
Types of variables in the theoretical model.

Figure 1 illustrates the mechanism of action of several variables in the theoretical model. The moderating effect is whether it will be affected by the moderator variable Z during the study of the influence of X on Y. There are obvious differences between industries with respect to the efficiency of investment by Chinese companies. This further implies that the impact of the level of green credit of enterprises on the innovation ability of enterprises may be unbalanced and insufficient due to differences in investment efficiency in industries and regions. Based on the different levels of investment, what is the impact of green credit on the green innovation capabilities of polluting companies? The effect of varying levels of investment efficiency of enterprises on green innovation capabilities is mainly reflected in the following two aspects.

First of all, in the process of market-oriented reforms, the degree of local government intervention in enterprises within its jurisdiction also shows obvious differences across industries, which will also affect the innovation behavior of enterprises. An important feature in regions and industries with relatively high investment efficiency is that local governments have less improper and excessive intervention in enterprises within their jurisdiction. Moreover, local governments can also provide more convenient institutional infrastructure conditions for innovative financing of enterprises within their jurisdiction. For example, building enterprise information databases, setting up regional policy innovation guidance funds, implementing scientific and technological innovation guidance plans, and introducing preferential fiscal and tax policies for scientific and technological innovation, etc. All of this helps to increase opportunities for companies to obtain external loans, boosting entrepreneurial innovation [49]. Conversely, local governments have more stringent qualifications for obtaining green credits for highly polluting companies in areas where the optimization and reform of the market-oriented business environment is relatively slow. Therefore, the uncertainty of external policies is greater, and local governments will restrict the innovation decisions of companies more. As a result of the adverse effects of various government policies, the risk of business innovation will be higher. Banks are reluctant to lend funds to companies with high risks and uncertain returns, which will hinder business innovation [50].

Secondly, the allocation of credit resources also shows obvious differences between sectors and regions. Banks in various areas have “relaxing forces”, and the distribution of credit resources will influence whether firms can obtain adequate credit financing support when carrying out investment activities [51]. Where the allocation of credit resources is more market-oriented, the autonomy of financial intermediaries such as banks is stronger. Banks are better equipped to gather and process business information, reducing the problem of information asymmetry between banks and businesses. To a certain extent, this contributes to a reduction in credit approval procedures and procedures for banks and other financial institutions. Consequently, it can effectively address the shortage of R&D funds and free up space for companies to improve investment efficiency [52]. Enhancing the effectiveness of investments further frees entrepreneurship from innovation and strengthens the enthusiasm for business innovation [34].

**Hypothesis** **2** **(H2).** *When firms’ investment efficiency is weak, the inhibiting effect of green credit policy on firms’ innovation capacity will be reinforced.*

#### 2.2.3. Green Credit, Corporate Debt Financing Costs and Corporate Green Innovation Capabilities

Supply factors in financial markets influence corporate funding decisions [29,53]. The rapid expansion of industries with high pollution and high energy consumption leads to the continuous increase in the discharge of industrial waste water and waste gas, which intensifies the deterioration of the environment and brings negative externalities. In the production process, the company harmed the external environment without compensation, i.e., the external diseconomy of production was produced [54]. In economic theory, common approaches to negative externalities include taxation and explicit property rights. That is, by taxing the non-economic external enterprise exactly at the marginal cost of the external enterprise or by defining property rights according to the Coase theorem. Nevertheless, it is difficult for the former to estimate the cost of externalities in monetary terms. In the latter case, the transaction cost cannot be zero in practice [55]. Implementing green credit requires banks to consider compliance with environmental testing standards as a significant precondition for credit approval in credit activities. This increases the borrowing threshold of the companies with high pollution and high energy consumption and can essentially be considered as a macro-economic policy tool to promote environmental protection that uses economic levers to guide environmental protection, internalizing the costs of corporate environmental pollution [56]. Government and the financial sector share information about business environmental protection, which helps to understand the relationship between environmental protection and financial credit. Negative signals could be sent to the “two high” enterprises, affecting their production and operation decisions and resource allocation. At the same time, it can send signals to the capital market to strengthen the management and supervision of corporate environmental information disclosure, thereby reducing the willingness of external creditors to provide debt capital. Following the “Green Credit Guidelines” formal implementation, high-polluting companies will face increased public pressure and moral condemnation, and they may even face risks of environmental disputes, causing external creditors to divest or turn down loan extensions. As a result, the level of debt financing of highly polluting companies is declining [31]. As shown in Figure 1, green credit can initially affect the cost to finance corporate debt, and then affect the green innovation ability of high-polluting companies by affecting the cost of financing corporate debt. The cost of debt funding may be used as an intermediary variable. Based on the foregoing analysis, this paper suggests the following assumption:

**Hypothesis** **3** **(H3).** *Green credit policy may increase the debt financing cost of enterprises and then reduce the green technology innovation of enterprises.*

## 3. Data and Methods

### 3.1. Data and Variables

This article mainly selects the data of China’s listed companies from 2010 to 2019. Based on the previous search objectives, this article selects the relevant data and tries to explain how policy works by analyzing mechanisms. In accordance with previous research objectives, this document selects relevant data. There are three data sources: First, the number of green invention patent applications mainly comes from the State Intellectual Property Office (http://pss-system.cnipa.gov.cn/sipopublicsearch/portal/uiIndex.shtml accessed on 9 December 2021). We specifically determine green patents based on the international patent classification in the “Green List of International Patent Classifications” issued by the World Intellectual Property Organization (WIPO) in 2010 (https://www.wipo.int/Classification/ipc/greeninventory/home accessed on 9 December 2021). Then, the financial data in the control variables, such as company size, asset-liability ratio, company life, ROA, proportion of tangible assets, cash holding rate, ownership concentration, earnings volatility, Tobin’s Q, stock return rate, and whether to disclose social responsibility report, are mainly from the CSMAR database.

The variable explained in this paper is the level of green innovation, which is represented by the natural log of the number of green patent applications. Although the selection of patents to measure the level of technological innovation has a certain degree of one-sidedness, patent data is still the only observable variable in the measure of technological innovation. Therefore, based on the study of [57], the number of patent licenses is selected as a surrogate indicator of enterprise technological innovation.

The explanatory variable of this article is the green credit policy. As for the definition of heavy polluting enterprises, [58] used the research to compare the Guidelines on Industry Classification of Listed Companies issued by the China Securities Regulatory Commission (CSRC) with the Classified Management Directory of Listed Companies’ Environmental Protection Verification Industry issued by the Ministry of Environmental Protection, so as to screen out samples of heavy polluting enterprises. It includes the mining industry, textile and garment fur industry, metal and nonmetal industry, petrochemical plastic industry, food and beverage industry, water, electricity and gas industry, biomedicine industry, paper making and printing industry, etc. By sampling highly polluting companies, we analyze the effect of green credit policy.

This paper selects investment efficiency as the moderator variable. Among them, the statistical method of the cost of debt financing is that of financial charges/total liabilities. The way of measuring investment efficiency is based on the measurement model described in [59]. This model has been widely used in the field of corporate finance as it allows the direct measurement of the investment efficiency of a given firm in a given year.

(1) $$Inveffi_{it}=\beta_{0}+\beta_{1}Invest_{i,t-1}+\beta_{2}Growth_{i,t-1}+\beta_{3}Lev_{i,t-1}+\beta_{4}Cash_{i,t-1}+\beta_{5}Age_{i,t-1}+\sum Ind+\sum Year+\varepsilon_{it}$$

Where $Inveffi_{it}$ is the amount of capital investment, which is equal to (capital expenditure + M&A expenditure-income from selling long-term assets-depreciation)/total assets; $Growth_{i,t-1}$ is equal to the growth rate of operating income; $Lev_{i,t-1}$ is the asset-liability ratio; $Cash_{i,t-1}$ is equal to cash and cash equivalents/Total assets; $Age_{i,t-1}$ is the listing period, which is equal to the natural logarithm of the company’s listing period. In addition, the model also controls the industry fixed effect Ind and the annual fixed effect Year. The residual of this formula is the investment efficiency. When the residual $\varepsilon_{it}$ > 0, it indicates that the company has overinvested. When the residual $\varepsilon_{it}$ < 0, it indicates that the enterprise has insufficient investment.

This paper takes debt financing cost as an intermediary variable, and the method of measuring debt financing cost is to divide the financial expenses of the enterprise by the total debt. In order to control the heterogeneous characteristics of the company, this article also uses the following control variables: company size, leverage level, company age, ROA, tangible asset ratio, cash holding ratio, equity concentration, whether to publish social responsibility reports and the degree of profit volatility, Tobin Q, stock yield. The specific variable definitions in the text are shown in Table 1.

In order to mitigate the influence of extreme values on empirical findings, this article winsorizes continuous variables that are less than 1% (or greater than 99%) of the quantile. To alleviate the endogenous problem, the control variables of the investment and financing equation (i.e., Equation (3)) in the regression analysis are all lagging one period (except for corporate age, equity concentration and corporate social responsibility). At the same time, the standard deviation of the test results is the adjustment of the cluster at the company level. Table 2 presents descriptive statistics of the main variables covered by the study.

### 3.2. Methods

The impact of green credit policy on the green technology innovation of heavily polluting companies is mainly achieved through political shocks. Specifically, first, the implementation of the green credit policy has reduced the ability of high-polluting companies to secure loans for green innovation. At the same time, high-polluting companies have spent more on reducing corporate pollution to cope with public monitoring of corporate environmental effects. To some degree, fewer funds are used to research companies’ green patents [60,61].

A higher level of green credit will raise the threshold for companies to access green credit funds and reduce green technology innovation among companies. On the contrary, as the level of innovation in green technologies decreases, it will be harder for companies to obtain green credit funds from banks. In this way, there is a mutual causality between the explanatory variable and the explained variable. This will establish a correlation between the explanatory variable and the error term, resulting in endogenous problems and inconsistent parameter estimates [62]. The policy is exogenous, and the difference-in-difference method may effectively mitigate the endogenous problem. Additionally, the DID model can not only control unobservable individual heterogeneity between samples, but also maintain the influence of unobservable demographic factors that change over time. Therefore, a consistent estimate of policy effects could be obtained. The DID method has these excellent properties and has been extensively used in policy assessment.

On this basis, we use the “Green Credit Guidelines” incident released by the China Banking Regulation Commission on 24 February 2012, as a political shock. Before the publication of the “Green Credit Guidelines” in 2012, credit policies of banking financial institutions paid little attention to green development issues. After 2012, banking financial institutions are required to clarify the direction and focus areas of green credit support. This paper uses the DID model to evaluate green credit guidelines’ impact on green technological innovation. The specific model is as follows:

(2) $$Greeninnovation_{it}=\alpha +\beta treated_{i}\times post_{t}+\gamma X_{it}+\delta_{i}+\lambda_{t}+\varepsilon_{it}$$

Among them, $Greeninnovation_{it}$_it refers to the number of green invention patent applications of a company (divided by the number of invention patent applications), and represents the company’s green innovation level. $treated_{i}$ is the group dummy variable, the treatment group company is 1, otherwise it is 0 (As companies in heavy polluting industries are directly affected by the green credit guidelines, they are treated as a treatment group and non-heavy polluting industries as a control group). $post_{t}$ is an event dummy variable, and the value is 1 in 2012 and later, otherwise the value is 0. $treated_{i}\times post_{t}$ are DID variables. $X_{it}$ includes a series of enterprise-level control variables. β is the coefficient of the interaction term, which measures the impact of green credit standards on green technological innovation. At the same time, $\delta_{i}$ is an individual fixed effect, and $\lambda_{t}$ is a time fixed effect to capture the influence of time-invariant enterprise-level factors and time-related factors.

In this paper, the impact of green credit on the technological innovation of heavily polluting enterprises is estimated by using the differential difference model of panel data. This paper further examines whether the moderating effect of investment efficiency exists. Therefore, based on model (2), investment efficiency is added as an intermediary variable.

(3) $$Greeninnovation_{it}=\alpha +\beta_{1}\left(treaed_{i}\times post_{t}\right)+\beta_{2}\left(treated_{i}\times post_{t}\times Efficiency_{it}\right)+\gamma X_{it}+\delta_{i}+\lambda_{t}+\varepsilon_{it}$$

Moreover, the implementation of green credit policies may indirectly impact green innovation capacities by altering the debt financing costs of high-polluting companies. In other words, the cost of debt finance has a mediating effect on developing the green innovation capacity of heavy polluting enterprises inhibited by green credit. As shown in Figure 1, the mediating effect means that the influence relationship between dependent variable and independent variable is not a direct causal chain relationship (X→Y), but an indirect influence is generated through some intermediate variable M (X→M→Y). This paper draws on the stepwise regression method proposed by [63] by constructing the following recursive model to test the mechanism of debt financing costs affecting the level of green innovation of urban enterprises. Among them, $\beta_{1}$ in formula 4 measures the total effect of green credit policy on the company’s green innovation capability. In formula 5, $\gamma_{1}$ measures the direct impact of green credit policies on the increase in debt financing costs of heavily polluting enterprises. And $\tau_{2}$ measures the mediating effect of debt financing costs, that is, the degree of indirect influence of green credit on enterprises’ level of green innovation of enterprises by changing the debt financing costs of heavily polluting enterprises.

(4) $$Greeninnovation_{it}=\beta_{0}+\beta_{1}D_{it}+\beta_{2}X_{it}+\delta_{i}+\lambda t+\varepsilon_{it}$$

(5) $$Debt_{it}=\gamma_{0}+\gamma_{1}D_{it}+\gamma_{2}X_{it}+\delta_{i}+\lambda t+\varepsilon_{it}$$

(6) $$Greeninnovation_{it}=\tau_{0}+\tau_{1}D_{it}+\tau_{2}Debt_{it}+\tau_{3}X_{it}+\delta_{i}+\lambda t+\varepsilon_{it}$$

## 4. Results

### 4.1. Basic Regression

This article uses the DID method to measure the impact of green credit on the level of corporate green innovation. At the same time, in order to determine whether the fixed-effect model or the random-effect model should be used, this paper uses the LM test and the Hausman test. The test results show that the two-way fixed-effects model should be used. Table 3, from left to right, represents the results of Pooled panel regression, individual random effect, individual fixed effect and bidirectional fixed effect, respectively.

Table 3 shows how green credit policies can limit companies’ green technological innovation. The DID coefficient is −0.07 and passed the 1% significance test. After implementing the green credit policy, banks will raise the loan threshold to improve their green rating. This leads to higher financing costs for polluters. Compared with non-green patents, green technology patents have higher technical requirements and more investment, which may not be substantially increased in the short term, but will decline.

### 4.2. Robustness Test

This paper used two methods to verify the accuracy of the conclusions. The first method was parallel trend testing. The second method was the placebo test.

#### 4.2.1. Parallel Trend Test

**Figure 2:**
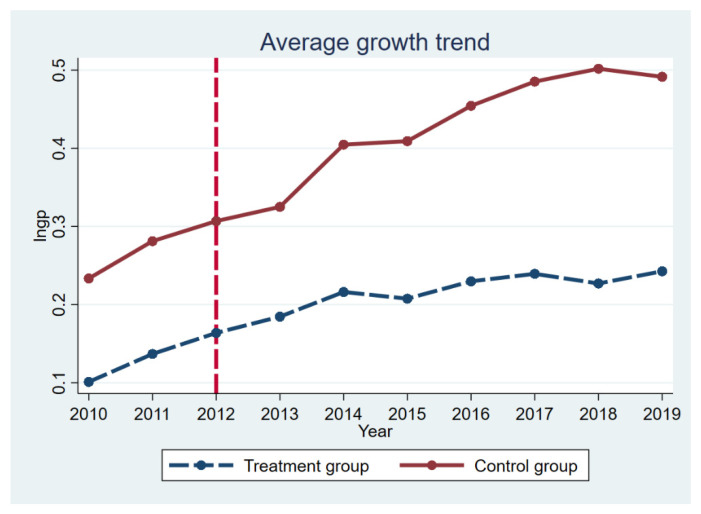
Parallel trend test.

The assumption of parallel trend is the premise of using the time-varying DID model, requiring the lngp of the treatment group and the control group to have the same changing direction before implementing the green credit policy. Therefore, this paper adopts the time trend diagram of the treatment group and the control group to conduct a parallel trend test, as shown in Figure 2. Before the policy time point, the trend of the average growth trend was basically parallel, but after the implementation of the policy, the gap gradually widened, indicating that the green credit policy is effective. Therefore, by plotting the time trend graph for the treatment and control groups, a rough conclusion can be drawn that addresses the parallel trend assumption. Before the implementation of the Green Credit Policy, the trend of growth in the levels of green patents of polluting and non-polluting enterprises was more or less the same. After implementing the green credit policy, the growth of the green innovation capacity of heavy-polluting enterprises was relatively slow. It is pointed out that the innovation capacity of heavy polluting enterprises may be hampered by the green credit policy.

#### 4.2.2. Placebo Test

**Figure 3:**
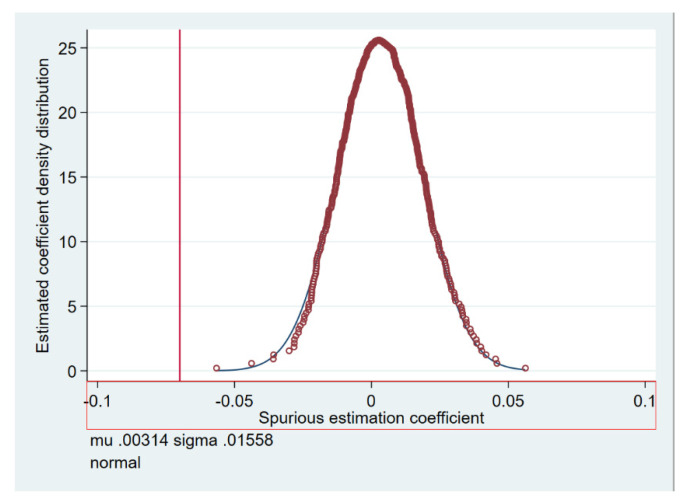
Placebo test.

The basic idea behind the double-difference placebo test is to estimate the duration of the dummy treatment group or dummy policy. If the coefficient of the “pseudo-political fictitious variable” remains significant in the fictitious situation, this means that the result of the initial estimation is likely to be biased. Changes in the level of green innovation of heavy pollutant companies may have been influenced by other political or random factors. This paper randomly selected the interaction term 500 times to check whether the coefficient is significantly different from the baseline estimation result and draws the distribution diagram of the regression coefficient. As shown in Figure 3, randomized coefficients are expected to follow a normal distribution and thus pass the placebo test.

### 4.3. Analysis of Heterogeneity

The nature and scope of company ownership may result in differences in the effectiveness of green credit policies. In general, compared to state-owned enterprises, non-state enterprises are less sensitive to environmental impacts in their day-to-day production and operations, and their consciousness of social responsibility is relatively weak. At the same time, enterprises of different sizes may have some differences in operational efficiency and financing channels. Therefore, differences in corporate ownership and size can lead to different characteristics of green innovation under the influence of green credit policies.

In order to explore the micro effects of green credit policies under different enterprise attributes, sample companies are divided into state-owned enterprises and non-state-owned enterprises according to different ownership attributes of enterprises. The sampled enterprises were divided into three quartiles based on differences in the size of the enterprise. The first third of the largest companies are defined as large companies, and the last two thirds are defined as small and medium-sized companies. The DID model is used to analyze the heterogeneous impact of green credit policies on green technological innovation. The empirical results are shown in the table below.

Table 4 shows that the heterogeneous impact of green credit policies on green innovation is mostly reflected in business property rights. Specifically, in the sub-sample regression of state-owned enterprises and non-state-owned enterprises, the coefficients of the interaction terms are −0.0599 and −0.0867, respectively, and the former does not pass the significance test. This shows that the inhibition effect of the green credit policy on the green innovation of heavily polluting enterprises is significant in the sample of non-state-owned enterprises, but not in the sample of state-owned enterprises. In the sub-sample regression of large enterprises and small and medium enterprises, the coefficients of the interaction terms are −0.167 and −0.0384, respectively, and neither of them has passed the significance test. This indicates that the policy inhibition effect of green credit on enterprises’ green innovation will not be more different due to the size of enterprises.

### 4.4. Mechanism Analysis

The results of Table 3 show that the implementation of green credit has hampered the ecological innovation capabilities of high-polluting companies. Concerning hypothesis 2, this article focuses on the moderating effect of investment efficiency and introduces the term interaction between investment efficiency and policy into the model. The moderating effect of investment efficiency is shown in the first column of Table 5. After introducing a regulated variable, the estimated coefficient of green credit policy is always significantly negative. The assessment of the coefficient of the adjustment variable shows that the coefficient of the interaction term between green credit policy and investment efficiency is significantly negative. The results show that the relationship between the implementation of green credit policies and the green innovation capabilities of heavily polluting companies will be negatively regulated by investment efficiency. Hypothesis 2 has been confirmed.

In order to further identify the internal mechanism of green credit policies, it is necessary to test the mediation effect of debt financing costs. According to the stepwise regression method, the models 4~6 are regressed. It can be seen from Table 5 that the coefficients of $\beta_{1}$,$\tau_{1}\;$and $\tau_{2}$ are all significant, so the intermediary variables are path dependent. Specifically, implementing green credit policies will lead to increased corporate debt costs. According to the procedure of the intermediary effect test, the intermediary effect of debt financing costs is −0.144. Green credit policy indirectly inhibits the level of green innovation capacity of firms by increasing the debt financing costs of highly polluting firms. At the same time, the estimated coefficient of the green credit policy is significantly negative under different models, which means that part of the intermediary effect of debt financing costs is significant. The above results indicate that the implementation of the green credit policy will increase the debt financing cost of heavily polluting companies, thereby inhibiting the development of the level of green innovation for heavily polluting companies. Hypothesis 3 has been confirmed.

After implementing green credit, financial institutions reduced the long-term debt of polluting companies to avoid risks. This will impede investment in R and D and the generation of business innovation. The implementation of green credit will harm the company’s green innovation. Mechanism analysis found that the credit constraints caused by the reduction of credit scale and the increase of credit costs are the main mechanism of action [11].

## 5. Discussion

This study makes a significant contribution to exploring the relationship between green credit policies and the green innovation capacity of highly polluting firms. Based on the implementation of green credit policies in China, this paper uses a difference-in-difference approach to identify policy impacts. This paper finds that the implementation of green credit policies inhibits the ability of heavily polluting firms to innovate green. This paper adopts a similar research methodology to [39], but the findings are quite different. Hong et al. [39] believes that the implementation of the green credit policy has improved the company’s green innovation capabilities. The research sample used in this article is not the same as that of the above research. This article focuses on heavy-polluting companies. The reason is that the current environmental pollution is mainly affected by polluting emissions from heavily polluting companies. However, this article does not consider that the green credit policy has no positive effect on heavily polluting companies. This article believes that the credit restraint effect and information transmission effect caused by policies reduce the credit resources and commercial credit available to enterprises, leading to a decline in the level of technological innovation. Moreover, this paper argues that the decline of green innovation may be triggered by the fact that it takes some time for the financial steering role of green credit and the resource allocation role of R&D investment to manifest. In the meantime, a study by [16] explains this point in a different light. It argues that switching social capital from polluting industries to non-polluting industries can facilitate green innovation in non-polluting industries, which in turn can lead to environmental improvements. That might be another explanation of the conclusions of that document.

In this paper, investment efficiency is considered the moderating variable and the cost of debt financing is considered the mediating variable in the analysis of the mechanism. Some existing studies concentrate on the unique impact of green credit policies. For example, Li et al. [64] argued that implementing green credit policies reduced the cost of debt financing to highly polluting companies. Zhang et al. [32] found that implementing green credit guidelines helps companies invest in renewable energy. These articles do not analyze the interactive effects that other influencing factors may have. This article integrates the impact of investment efficiency and debt financing costs into the mechanism analysis of this article, trying to reveal the mechanism of green credit inhibiting technological innovation of polluting enterprises. The results of this article show that investment efficiency has not played a moderating role. Wang et al. [65] pointed out that green credit can improve the investment efficiency of enterprises. However, the research results of this article do not confirm that the improvement of investment efficiency will affect the effect of green credit policy on the level of green technology innovation. Moreover, the results show that an increase in the cost of debt financing can make policy implementation more effective. The results of this study are the same as those obtained by [56]. Both articles argue that the implementation of green credit policies will lead to an increase in the cost of debt financing and thus less funding for green innovation by heavy polluters.

In the meantime, this article breaks down the research sample into small and medium-sized enterprises (SMEs) and large enterprises. This article argues that the disincentive effect of green credit on corporate green innovation does not differ by company size. This differs from the conclusions reached by [66]. Singh et al. [66] uses environmental performance as the explained variable, and the research shows that the effects of green policies will vary depending on the size of the company. This paper argues that company size is not a critical factor in determining whether green policies are effective. At the same time, the research results of this article conclude that green credit is significant in the sample of non-state-owned enterprises, but not in the sample of state-owned enterprises. This is the same as the conclusion of [67], who believes that compared with private enterprises, state-owned enterprises have more financing channels. Therefore, the restrictions of green credit policies will have a greater impact on private enterprises.

There are several limitations to this study. Firstly, since the enactment of the green credit policy, it has been more difficult for the heavily polluting enterprises to access social capital. Therefore, it is worth considering whether this social capital has gone into non-heavily polluting enterprises and improved environmental benefits. This is a direction that may require further consideration going forward. Then, the ability of companies to innovate in green is only one way for them to reduce their pollution levels. Innovation is not the ultimate goal. Reducing pollution is a desired result of green credit policies. Future research will focus on indicators that represent the level of pollution reduction of high-polluting enterprises. However, due to the complexity of the current corporate pollution reduction indicators, there is no uniformity. As a result, data collection is more difficult.

## 6. Conclusions

Based on the panel data of China’s A-share non-financial listed companies from 2010 to 2019, this paper constructs a DID model to study the impact of green credit guidelines on the green innovation capabilities of heavily polluting companies. With respect to the impact of environmental regulation on business technological innovation, Porter’s weak hypothesis remains controversial. This study uses Chinese listed companies as a sample to empirically test the inhibitory effect of green credit standards on companies’ green innovation capabilities, which are manifested in three aspects: (1) Under the green credit policy, it is difficult for heavily polluting enterprises to obtain more credit support and better loan interest rates for a short time, leading to difficulty for enterprises to have enough funds to research green innovative products and technologies, (2) The implementation of green credit policy leads to the restriction of green credit from commercial banks for heavily polluting enterprises, which stimulates enterprises to increase commercial credit instead of debt financing. However, due to the differences in the ability of different heavy polluting enterprises to obtain commercial credit, many heavy polluting enterprises find it difficult to obtain enough funds to supplement the credit gap of commercial banks [68], (3) Pilot policies are a gradual process, and green projects are indeed characterized by long cycles, high risks and high evaluation costs. There is a gradual change in the attitude and evaluation of financial institutions towards green projects. The support for green projects is obviously supported by the amount of financial resources, but there is no preferential financing cost [69].

## Figures and Tables

**Table 1:** Variable description.

Variable	Statistic	Description
Green innovation level	lngp	Ln (1 + Number of green patent applications)
Group dummy variable	treated	Heavy polluting enterprise = 1; Non-heavy polluting enterprises = 0
Event dummy variable	post	The value for 2012 and later is 1. Otherwise, the value is 0
Investment efficiency	inveffi	Measured by Richardson’s (2006) model
Debt financing cost	debt	Total financial expenses/liabilities
Company size	size	The natural log of total assets at year end
Leverage	lev	Asset-liability ratio
Company age	age	The number of years the company has been listed
Return on assets	roa	Net profit/Total average assets
Proportion of tangible assets	tar	(Owners’ equity − Intangible Assets − Deferred Assets)/Total assets amount
Cash holding ratio	cash	Cash and cash equivalents ending balance/current liabilities
Ownership concentration	equity	Shareholding ratio of the company’s largest shareholder
Social responsibility	public	Public social responsibility report = 1; Non-disclosure of social responsibility report = 0
Earning volatility	std	Standard deviation of return on assets in years t−3 to T
Tobin’s Q	tq	(Market value of tradable shares + par value of non-tradable shares)/(Total Assets − net intangible assets − net goodwill)
Return on stock	ret	Annual return on individual shares

**Table 2:** Descriptive statistics.

Variable	Obs	Mean	Std. Dev.	Min	Max
lngp	12,378	0.2871725	0.7132743	0	6.590301
po	12,420	0.5273752	0.4992701	0	1
after	12,420	0.8	0.4000161	0	1
size	12,419	22.24249	1.331667	14.75859	28.63649
lev	12,419	0.4632725	0.6146905	0.0070799	29.69759
age	12,420	2.793565	0.397153	0.6931472	3.688879
roa	12,419	0.0506745	0.3824283	−28.94023	22.00289
tar	12,419	0.9304783	0.079834	0.317304	1
cash	12,419	0.1745579	0.1345231	0.0001508	0.9147874
equity	12,420	34.32527	15.02518	0.2863	89.9858
public	12,368	0.3036061	0.4598331	0	1
std	12,420	0.0949768	4.084318	0.0006314	453.1186
tq	12,048	2.164018	2.746482	0.683714	122.1895
ret	12,410	435.1414	39555.46	−353.706	4324004
inveffi	11,014	0.0412461	0.0512885	4.69 × 10^−6^	0.4645226
debt	12,420	0.0111637	0.1502056	−2.454517	14.79254

**Table 3:** Basic return.

	(1)	(2)	(3)	(4)
**did**	−0.188 ***	−0.028	−0.002	−0.070 ***
	[0.013]	[0.017]	[0.017]	[0.027]
**size**	0.203 ***	0.128 ***	0.079 ***	0.062 ***
	[0.010]	[0.016]	[0.017]	[0.017]
**lev**	−0.01	0.007	0.006	0.017
	[0.014]	[0.010]	[0.010]	[0.011]
**age**	−0.076 ***	0.068 **	0.173 ***	−0.113
	[0.016]	[0.032]	[0.044]	[0.082]
**roa**	−0.011	0.009	0.008	0.016
	[0.015]	[0.007]	[0.007]	[0.010]
**tar**	0.229 ***	0.213 **	0.155	0.147
	[0.069]	[0.099]	[0.100]	[0.100]
**cash**	0.325 ***	0.041	0.034	0.026
	[0.052]	[0.056]	[0.057]	[0.058]
**equity**	−0.004 ***	−0.001	0	0
	[0.001]	[0.001]	[0.001]	[0.001]
**public**	0.093 ***	0.047 **	0.022	0.015
	[0.015]	[0.023]	[0.026]	[0.026]
**std**	−0.008	0.008	0.008	0.007
	[0.010]	[0.015]	[0.014]	[0.013]
**tq**	0.015 ***	0.007 ***	0.004 **	0.003
	[0.003]	[0.003]	[0.002]	[0.002]
**ret**	0	0	0	0
	[0.000]	[0.000]	[0.000]	[0.000]
**_cons**	−4.149 ***	−2.936 ***	−2.102 ***	−0.888 **
	[0.233]	[0.375]	[0.385]	[0.441]
**Year-fixed effect**	Control	Control	Control	Control
**Firm-fixed effect**	Control	Control	Control	Control
**N**	12048	12048	12048	12048
**R-squared**	0.136	0.107	0.703	0.705

Notes: *** and ** indicate significant at the 1% and 5% levels, respectively.

**Table 4:** Heterogeneity analysis.

	(5)	(6)	(7)	(8)
	State-Owned	Non-State-Owned	Large Firms	Small Firms
**did**	−0.0599	−0.0867 **	−0.167	−0.0384
	(−1.32)	(−2.67)	(−1.94)	(−1.72)
**size**	0.0388	0.0842 ***	0.206 **	0.0580 ***
	−1.44	−3.62	−3.21	−3.47
**lev**	0.0763	0.0204	−0.0623	0.0104
	−1.1	−1.9	(−0.50)	−1
**age**	−0.237	−0.0262	−0.38	0.007
	(−0.78)	(−0.34)	(−1.25)	−0.11
**roa**	0.0628	0.0183	0.285	−0.0048
	−1.44	−1.45	−1.47	(−0.59)
**tar**	−0.0126	0.166	−0.133	0.0231
	(−0.05)	−1.42	(−0.38)	−0.28
**cash**	−0.14	0.0276	−0.0376	0.0418
	(−1.20)	−0.44	(−0.20)	−0.77
**equity**	−0.00117	−0.00098	−0.00066	−9.2 × 10^−5^
	(−0.72)	(−0.86)	(−0.35)	(−0.10)
**public**	0.00318	0.0128	−0.0509	0.0445
	−0.09	−0.36	(−1.30)	−1.52
**std**	0.0384	−0.0003	0.0361	−0.03
	−1.63	(−0.07)	−1.61	(−1.84)
**tq**	−0.00073	0.00538 *	0.0372	0.00196
	(−0.17)	−2.03	−1.59	−1.28
**ret**	−7.35× 10^−8^ ***	0.000000591 ***	0.000139	−3.62 × 10^−8^
	(−11.23)	−22.11	−0.91	(−1.21)
**_cons**	0.218	−1.663 **	−3.052	−1.120 **
	−0.18	(−3.12)	(−1.77)	(−2.60)
**Year-fixed effect**	Control	Control	Control	Control
**Firm-fixed effect**	Control	Control	Control	Control
**N**	5483	6153	5094	6861
**R-squared**	0.1405	0.1356	0.1963	0.1357

Notes: ***, ** and * indicate significant at the 1%, 5% and 10% levels, respectively.

**Table 5:** Mechanism analysis.

	(9)	(10)	(11)	(12)
	Regulating Effect	Mediating Effect		
**did**	−0.206 ***	−0.188 ***	0.00237 *	−0.188 ***
	(−13.58)	(−14.71)	(−2.26)	(−14.69)
**did_inveffi**	0.321			
	−1.83			
**debt**				−0.144 **
				(−3.49)
**size**	0.224 ***	0.203 ***	0.00125	0.204 ***
	−18.79	−20.42	−1.89	−20.44
**lev**	−0.115 **	−0.01	0.0244 ***	−0.00651
	(−2.72)	(−0.69)	−3.33	(−0.46)
**age**	−0.114 ***	−0.0757 ***	0.00196	−0.0754 ***
	(−6.02)	(−4.77)	−1.19	(−4.75)
**roa**	−0.436 ***	−0.0109	0.00913	−0.00963
	(−5.62)	(−0.75)	−1.09	(−0.68)
**tar**	0.320 ***	0.229 ***	0.00712	0.230 ***
	−4.2	−3.33	−1.16	−3.34
**cash**	0.452 ***	0.325 ***	−0.176 ***	0.300 ***
	−6.14	−6.23	(−14.87)	−5.43
**equity**	−0.00347 ***	−0.00359 ***	−0.0000696 *	−0.00360 ***
	(−6.30)	(−7.07)	(−2.22)	(−7.09)
**public**	0.0844 ***	0.0929 ***	−0.00258 *	0.0925 ***
	−5.31	−6.12	(−2.20)	−6.09
**std**	−0.337 ***	−0.00774	−0.00256	−0.00811
	(−4.88)	(−0.76)	(−0.67)	(−0.81)
**tq**	0.0257 ***	0.0154 ***	−0.000323	0.0154 ***
	−7.11	−5.19	(−1.14)	−5.2
**ret**	1.62 × 10^−8^	8.47 × 10^−9^	1.36 × 10^−9^	8.67 × 10^−9^
	−0.89	−0.39	−1.23	−0.4
**_cons**	−4.543 ***	−4.149 ***	−0.00839	−4.151 ***
	(−16.61)	(−17.82)	(−0.48)	(−17.83)
**Year-fixed effect**	Control	Control	Control	Control
**Firm-fixed effect**	Control	Control	Control	Control
**N**	10770	12048	12048	12048
**R-squared**	0.1405	0.1356	0.1963	0.1357

Notes: ***, ** and * indicate significant at the 1%, 5% and 10% levels, respectively.

## Data Availability

Not applicable.

## References

[1] Leichenko R., O’Brien K. (2018). Environmental Change and Globalization: Double Exposures.

[2] Schiederig T., Tietze F., Herstatt C. (2012). Green innovation in technology and innovation management—An exploratory literature review. Rd Manag..

[3] Azzone G., Bertelè U., Noci G. (1997). At last we are creating environmental strategies which work. Long Range Plan..

[4] Wu H.G., Wang S.H. (2016). Do bond market participants pay attention to corporate environmental information?—Empirical evidence from China’s heavily polluting listed companies. Account. Res..

[5] Wang K., Zhang X. (2021). The effect of media coverage on disciplining firms’ pollution behaviors: Evidence from Chinese heavy polluting listed companies. J. Clean. Prod..

[6] Rennings K. (2000). Redefining innovation—Eco-innovation research and the contribution from ecological economics. Ecol. Econ..

[7] Andersen D.C. (2017). Do credit constraints favor dirty production? Theory and plant-level evidence. J. Environ. Econ. Manag..

[8] Porter M.E., Van der linde C. (1995). Toward a New Conception of the Environment-Competitiveness Relationship. J. Econ. Perspect..

[9] Li Z., Liao G., Albitar K. (2020). Does corporate environmental responsibility engagement affect firm value? The mediating role of corporate innovation. Bus. Strategy Environ..

[10] Palmer K., Oates W.E., Portney P.R. (1995). Tightening Environmental Standards: The Benefit-Cost or the No-Cost Paradigm?. J. Econ. Perspect..

[11] Caragnano A., Mariani M., Pizzutilo F., Zito M. (2020). Is it worth reducing GHG emissions? Exploring the effect on the cost of debt financing. J. Environ. Manag..

[12] Ling S., Han G., An D., Hunter W.C., Li H. (2021). The impact of green credit policy on corporate green innovation. Stud. Sci. Sci..

[13] He L., Liu R., Zhong Z., Wang D., Xia Y. (2019). Can green financial development promote renewable energy investment efficiency? A consideration of bank credit. Renew. Energy.

[14] He D., Zhang X. (2007). Thoughts on several issues in the promotion of green credit by Chinese commercial banks. Shanghai Financ..

[15] Guo P. (2014). Financial policy innovation for social change: A case study of China’s green credit policy. Int. Rev. Sociol..

[16] Xu L. (2013). On the evaluation of performance system incorporating “green credit” policies in China’s financial industry. J. Financ. Risk Manag..

[17] Zhang K., Li Y., Qi Y., Shao S. (2021). Can green credit policy improve environmental quality? Evidence from China. J. Environ. Manag..

[18] Lei X., Wang Y., Zhao D., Chen Q. (2021). The local-neighborhood effect of green credit on green economy: A spatial econometric investigation. Environ. Sci. Pollut. Res..

[19] Zhang S., Wu Z., Wang Y., Hao Y. (2021). Fostering green development with green finance: An empirical study on the environmental effect of green credit policy in China. J. Environ. Manag..

[20] Wang M., Liao G., Li Y. (2021). The relationship between environmental regulation, pollution and corporate environmental responsibility. Int. J. Environ. Res. Public Health.

[21] Oosterhaven J., Broersma L. (2007). Sector structure and cluster economies: A decomposition of regional labour productivity. Reg. Stud..

[22] Xu S., Zhao X., Yao S. (2018). Analysis of the effect of green credit on the upgrading of industrial structure. J. Shanghai Univ. Financ. Econ..

[23] Shao C., Wei J., Liu C. (2021). Empirical analysis of the influence of green credit on the industrial structure: A case study of China. Sustainability.

[24] He L., Wu C., Zhong Z., Zhu J. (2018). Green credit, internal and external policies, and commercial bank competitiveness: Based on an empirical study of 9 listed commercial banks. Financ. Econ. Res..

[25] King R.G., Levine R. (1993). Finance and growth: Schumpeter might be right. Q. J. Econ..

[26] Luo S., Yu S., Zhou G. (2021). Does green credit improve the core competence of commercial banks? Based on quasi-natural experiments in China. Energy Econ..

[27] Yin X. (2021). Research on the impact of green credit on the financial performance of commercial banks. Financ. Mark..

[28] Liu J.-Y., Xia Y., Fan Y., Lin S.-M., Wu J. (2017). Assessment of a green credit policy aimed at energy-intensive industries in China based on a financial CGE model. J. Clean. Prod..

[29] Lemmon M., Roberts M.R. (2010). The response of corporate financing and investment to changes in the supply of credit. J. Financ. Quant. Anal..

[30] Li W., Cui G., Zheng M. (2021). Does green credit policy affect corporate debt financing? Evidence from China. Environ. Sci. Pollut. Res..

[31] Peng B., Yan W., Elahi E., Wan A. (2021). Does the green credit policy affect the scale of corporate debt financing? Evidence from listed companies in heavy pollution industries in China. Environ. Sci. Pollut. Res..

[32] Zhang K., Wang Y., Huang Z. (2021). Do the green credit guidelines affect renewable energy investment? Empirical research from China. Sustainability.

[33] Yang Z., Fang H. (2020). Research on green productivity of chinese real estate companies—Based on SBM-DEA and TOBIT models. Sustainability.

[34] Zhang M., Xu H., Feng T. (2019). Business environment, relational lending and technological innovation of small and medium-sized enterprises. J. Shanxi Univ. Financ. Econ..

[35] Lai X., Yue S., Chen H. (2021). Can green credit increase firm value? Evidence from Chinese listed new energy companies. Environ. Sci. Pollut. Res..

[36] Yao S., Pan Y., Sensoy A., Uddin G.S., Cheng F. (2021). Green credit policy and firm performance: What we learn from China. Energy Econ..

[37] Ling S., Han G., An D., Hunter W.C., Li H. (2020). The impact of green credit policy on technological innovation of firms in pollution-intensive industries: Evidence from China. Sustainability.

[38] Hao F., Xie Y., Liu X. (2020). The impact of green credit guidelines on the technological innovation of heavily polluting enterprises: A quasi-natural experiment from China. Math. Probl. Eng..

[39] Hong M., Li Z., Drakeford B. (2021). Do the green credit guidelines affect corporate green technology innovation? Empirical research from China. Int. J. Environ. Res. Public Health.

[40] Liu S., Xu R., Chen X. (2021). Does green credit affect the green innovation performance of high-polluting and energy-intensive enterprises? Evidence from a quasi-natural experiment. Environ. Sci. Pollut. Res..

[41] Hu G., Wang X., Wang Y. (2021). Can the green credit policy stimulate green innovation in heavily polluting enterprises? Evidence from a quasi-natural experiment in China. Energy Econ..

[42] Guo Q., Zhou M., Liu N., Wang Y. (2019). Spatial effects of environmental regulation and green credits on green technology innovation under low-carbon economy background conditions. Int. J. Environ. Res. Public Health.

[43] Rubashkina Y., Galeotti M., Verdolini E. (2015). Environmental regulation and competitiveness: Empirical evidence on the Porter Hypothesis from European manufacturing sectors. Energy Policy.

[44] Whaller N., Whitehead B. (1994). It’s not easy being green. Harv. Bus. Rev..

[45] Leonard H.J. (2006). Pollution and the Struggle for the World Product: Multinational Corporations, Environment, and International Comparative Advantage.

[46] Yuan B., Xiang Q. (2018). Environmental regulation, industrial innovation and green development of Chinese manufacturing: Based on an extended CDM model. J. Clean Prod..

[47] Kneller R., Manderson E. (2012). Environmental regulations and innovation activity in UK manufacturing industries. Resour. Energy Econ..

[48] Shi B., Feng C., Qiu M., Ekeland A. (2018). Innovation suppression and migration effect: The unintentional consequences of environmental regulation. China Econ. Rev..

[49] Feng T., Zhang M. (2020). Business environment, financial development and enterprise technological innovation. Technol. Prog. Countermeas..

[50] Zadeh M.H., Magnan M., Cormier D., Hammami A. (2021). Environmental and social transparency and investment efficiency: The mediating effect of analysts’ monitoring. J. Clean. Prod..

[51] Chava S., Oettl A., Subramanian A., Subramanian K.V. (2013). Banking deregulation and innovation. J. Financ. Econ..

[52] Arrow K.J., Hurwicz L. (1962). Competitive stability under weak gross substitutability: Nonlinear price adjustment and adaptive expectations. Int. Econ. Rev..

[53] Faulkender M., Petersen M.A. (2006). Does the source of capital affect capital structure?. Rev. Financ. Stud..

[54] Randall A. (1972). Market solutions to externality problems: Theory and practice. Am. J. Agric. Econ..

[55] Meade J.E. (1973). The Theory of Economic Externalities: The Control of Environmental Pollution and Similar Social Costs.

[56] Dong Q. (2018). The impact of technological finance on the total factor productivity of city commercial banks—Based on the perspective of moderating effect and mediating effect. Financ. Theory Pract..

[57] Qi S., Lin S., Cui J. (2018). Can the environmental rights trading market induce green innovation?—Evidence based on green patent data of Chinese listed companies. Econ. Res..

[58] Shen H., Ma Z. (2014). Regional economic development pressure, corporate environmental performance and debt financing. Financ. Res..

[59] Richardson S. (2006). Over-investment of free cash flow. Rev. Account. Stud..

[60] Yang X., Yao Y. (2012). Environmental compliance and firm performance: Evidence from China. Oxf. Bull. Econ. Stat..

[61] Hamamoto M. (2006). Environmental regulation and the productivity of Japanese manufacturing industries. Resour. Energy Econ..

[62] Antonakis J., Bendahan S., Jacquart P., Lalive R. (2010). On making causal claims: A review and recommendations. Leadersh. Q..

[63] Baron R.M., Kemmy D.A. (1986). The moderator mediator variable distinction in social psychological research. J. Pers. Soc. Psychol..

[64] Li C., Lu N., Li D., Wang X. (2021). Corporate green innovation: Government regulation, information disclosure and investment strategy evolution. Stud. Sci. Sci..

[65] Wang Y.L., Lei X.D., Long R.Y. (2021). Can green credit policy promote the corporate investment efficiency?. China Popul. Resour. Environ..

[66] Singh S.K., Del Giudice M., Chierici R., Graziano D. (2020). Green innovation and environmental performance: The role of green transformational leadership and green human resource management. Technol. Forecast. Soc. Chang..

[67] Yu B. (2021). How Green Credit Policy Affects Technological Innovation of Heavy-polluting Enterprises?. Econ. Manag..

[68] Chen X., Shi Y., Song X. (2019). Green credit constraints, commercial credit and corporate environmental governance. Int. Financ. Stud..

[69] Li R., Liu L. (2021). Green finance and green innovation of enterprises. J. Wuhan Univ. Philos. Soc. Sci..

